# An auto-inducible phosphate-controlled expression system of *Bacillus licheniformis*

**DOI:** 10.1186/s12896-018-0490-6

**Published:** 2019-01-09

**Authors:** Nguyen Thanh Trung, Nguyen Minh Hung, Nguyen Huy Thuan, Nguyen Xuan Canh, Thomas Schweder, Britta Jürgen

**Affiliations:** 1 Center for Molecular Biology, Institute of Research and Development, Duy Tan University, Danang, Vietnam; 2 Faculty of Biotechnology, Vietnam National University of Agriculture, Hanoi, Vietnam; 3 Pharmaceutical Biotechnology, Institute of Pharmacy, University Greifswald, Greifswald, Germany

**Keywords:** *Bacillus licheniformis*, Heterologous gene expression, Phosphate starvation, Phytate

## Abstract

**Background:**

A promoter that drives high-level, long-term expression of the target gene under substrate limited growth conditions in the absence of an artificial inducer would facilitate the efficient production of heterologous proteins at low cost. A novel phosphate-regulated expression system was constructed using the promoter of the phytase encoding gene *phyL* from *Bacillus licheniformis* for the overexpression of proteins in this industrially relevant host.

**Results:**

It is shown that the *phyL* promoter enables a strong overexpression of the heterologous genes *amyE* and *xynA* in *B. licheniformis* when cells were subjected to phosphate limitation. Whether *B. licheniformis* can use phytate as an alternative phosphate source and how this substrate influences the P*phyL* controlled gene expression under growth conditions with limited inorganic phosphate concentrations were also investigated. It is shown that *B. licheniformis* cells are able to use sodium phytate as alternative phosphate source. The addition of small amounts of sodium phytate (≤ 5 mM) to the growth medium resulted in a strong induction and overexpression of both model genes in *B. licheniformis* cells under phosphate limited growth conditions.

**Conclusions:**

The P*phyL* controlled expression of the investigated heterologous genes in *B. licheniformis* is strongly auto-induced under phosphate limited conditions. The proposed P*phyL* expression system enables an overexpression of target genes in *B. licheniformis* under growth conditions, which can be easily performed in a fed-batch fermentation process.

**Electronic supplementary material:**

The online version of this article (10.1186/s12896-018-0490-6) contains supplementary material, which is available to authorized users.

## Background

*Bacillus licheniformis* is a saprophytic bacterium that is generally recognized as safe (GRAS) by the U.S. Food and Drug Administration [1]. The ability to produce and secrete high amounts of proteins into the extracellular medium (20-25 g/L) makes this bacterium to one of the most important industrial hosts for the large-scale production of industrial enzymes, such as amylases, proteases, phytases, and other specialty enzymes [2]. Another advantage of *B. licheniformis* is its ability to grow rapidly in simple media to high-cell-densities, which is favourable for an industrial-scale production.

The expression systems used in *B. licheniformis* were mostly developed for *B. subtilis*. At present, three types of expression systems that contain (i) constitutive promoters, (ii) inducer-specific promoters and (iii) auto-inducible promoters have been used for high-level production of heterologous proteins in *Bacillus subtilis* (e.g., [3–5]). Among them, expression systems containing inducer-specific promoters (e.g., P*spac* and P*xyl*) are the most widely used type. However, the requirement for specific inducers, such as IPTG or xylose, increases the cost of their large-scale application [6, 7]. Constitutive expression systems allow for continuous transcription of their target gene, and thus, are not suitable for the production of potential toxic proteins. In contrast, auto-inducible expression systems that require no specific inducers are ideal for the industrial production of heterologous proteins at low cost. These expression systems are induced by a variety of environmental factors, which can be easily simulated in industrial fermentation processes [8]. For example, nutrient limitation, e.g. glucose, is such a suitable signal for the induction of an auto-inducible promoter system [9]. Furthermore, an expression system using the phosphate starvation inducible *pst* promoter has been developed for *B. subtilis* [10]. However, a comparable auto-inducible promoter system has so far not been shown for *B. licheniformis*.

It has been recently demonstrated that phosphate starvation conditions induce a tightly regulated set of genes, which are involved in the mobilization of alternative phosphate sources by *B. licheniformis* cells. Among them, the phytase PhyL belongs to the most abundant extracellular protein under these conditions [11]. Phytase is an enzyme that catalyses the hydrolysis of phytate, the salt of phytic acid, to release a series of *myo*-inositol phosphate intermediates and inorganic phosphate (Pi) [12, 13]. Phytate is the major storage form of phosphate in plant seeds such as cereal and oilseeds (1 to 5% by weight) [14]. The strong expression of the *phyL* gene indicated that *B. licheniformis* cells might use phytate as an alternative phosphate source when concentration of Pi becomes limiting [11, 15]. It could be therefore concluded that the P*phyL* promoter would be a good candidate for the construction of a novel expression system that can be used for the production of heterologous proteins in *B. licheniformis* under phosphate limited growth conditions.

In this study, the suitability of the *phyL* promoter as a novel auto-inducible phosphate-regulated expression system for *B. licheniformis* was investigated by means of translational reporter gene fusions with the heterologous genes *amyE* and *xynA* from *B. subtilis* both at the transcriptional and translational level. Furthermore, the role of phytate as an alternative, natural phosphate source for the growth of *B. licheniformis* cells and as an inducer for the expression of the *phyL* promoter were studied.

## Methods

### Strains and cultivation

All bacterial strains used in this study are listed in Table 1. *B. licheniformis* MW3 (*B. licheniformis* DSM13 (*ΔhsdR1*, *ΔhsdR2*)), which ensures high transformation efficiencies due to the deletion of two type I restriction modification systems *hsdR1* and *hsdR2*, was used as the host strain [16]. Belitzky minimal medium (BMM) was used in all growth experiments [17]. The cells were cultivated at 37^ο^C with 200 rpm in 200 mL BMM (pH 7.0) containing 15 mM (NH_4_)_2_SO_4_, 8 mM MgSO_4_·7H_2_O, 27 mM KCl, 7 mM sodium citrate dihydrate, and 50 mM Tris-HCl (pH 7.5) supplemented with 0.6 mM KH_2_PO_4_, 2 mM CaCl_2_·2H_2_O, 1 μM FeSO_4_·7H_2_O, 10 μM MnSO_4_·4H_2_O, and 11 mM glucose. For the phosphate starvation experiments, the concentration of phosphate was reduced to 0.15 mM KH_2_PO_4_.

**Table 1:** Bacterial strains and plasmids used in this study

Strains or plasmids	Relevant genotype	Reference
Strains		
*E. coli* DH10B	F−, *mrcA*, Δ(*mrr-hsdRMS-mrcBC*), *Φ80dlacZ,* Δ*M15,* Δ*lacX74, deoR, ecA1,endA1,araD139,* Δ*(ara,leu)7697, galU, galK, λ−, nrspL, nupG*	Gibco BRL
*B. licheniformis* MW3	Δ*hsdR1,* Δ*hsdR2*	[16]
*B. licheniformis* TH3	Δ*hsdR1,* Δ*hsdR2,* pKUC3	This study
*B. licheniformis* TH4	Δ*hsdR1,* Δ*hsdR2,* pKUC4	This study
Plasmids		
pKUC	shuttle vector based on pUC18 and pKTH290	[19]
pKUC3	pKUC containing the PphyL′-′amyE fusion	This study
pKUC4	pKUC containing the PphyL′-′xynA fusion	This study

### Construction of strains

The activity of the P*phyL* promoter was analyzed by means of translational reporter gene fusions. For this purpose, an approximately 300-bp fragment containing the P*phyL* promoter from *B. licheniformis* DSM13 (Additional file 1: Figure S2) was cloned in front of the α-amylase and xylanase reporter genes from *B. subtilis* 168 with the primer pairs 1/2 and 1/5, respectively (Additional file 1: Table S1). The *amyE* and *xynA* genes from *B. subtilis* 168 were amplified with the primer pairs 3/4 and 6/7, respectively (Additional file 1: Table S1). The P*phyL*′-′*amyE* and P*phyL′-′xynA* fusions were constructed by means of the precise gene fusion polymerase chain reaction strategy described by Yon and Fried [18] by using the primer pairs 1/4 and 1/7, respectively. The PCR-fusion fragments were then inserted into the *Xba*I and *Kpn*I sites of the multi-copy plasmid pKUC (this shuttle vector is based on the pUC18 and pKTH290 plasmids [19]) resulting in vector pKUC3 and pKUC4, respectively. These vectors were used to transform the *B. licheniformis* strain MW3 by electroporation [20] resulting in the strains TH3 (pKUC3) and TH4 (pKUC4), respectively. These strains were then cultivated in phosphate-limited BMM as already described. Culture supernatants for enzyme assays were taken at different time points during the cultivation. The first sample was taken during the exponential growth phase (four hours after cultivation), the second sample during the transient phase and additional samples were taken 2, 4, 6, 8, 10, 12 and 14 h after onset of the stationary growth phase.

### Enzyme assays

Activity of the amylase AmyE was determined with the Ceralpha kit (Megazyme International Ireland Ltd., Bray, Ireland). Amylase activity was calculated in “international units” (IU) by the equation: IU/mL = 4.6 x (ΔE_400_ × 4.7 x Dilution) (ΔE_400_ = Absorbance at 400 nm (reaction) – Absorbance at 400 nm (blank)). One international unit of activity is defined as the amount of enzyme required to release one micromole of glucose-reducing sugar equivalents per minute under defined conditions of temperature and pH (40 °C, pH 6.5) [21].

Activity of the xylanase XynA was measured using the modified dinitrosalicylic acid (DNSA) method [22] with some modifications as described in details by Nguyen et al. [23]. One international unit (IU/mL) of xylanase activity was defined as the amount of enzyme that liberates one micromole of reducing sugar equivalent to xylose per minute under the assay conditions described.

### Analysis of the extracellular proteins

The proteins in the supernatant samples were separated by one-dimensional (1D-) SDS-PAGE. In brief, 20 μL of supernatant samples were mixed with 5 μL SDS sample buffer (50 mM Tris-HCl (pH 6.8), 2% SDS, 10% glycerol, 0.1% bromphenol blue) and denatured at 90 °C for 10 min. The SDS-PAGE gel included a separating gel (10% “Acrylamide-Solution (30%)-Mix 37.5:1” (Bio-Rad, USA), 0.4 M Tris (pH 8.8), 0.1% SDS, 0.1% APS, 0.04% TEMED) and a stacking gel (4% “Acrylamide-Solution”, 0.125 M Tris (pH 6.8), 0.1% SDS, 0.05% APS, 0.1% TEMED). The protein separation according to their molecular weight was conducted at 150 V for one hour using a Protean II Cell system (BIO-RAD). After electrophoresis, the gel was stained with Coomassie Brilliant Blue R-250.

### Growth analysis

The growth of *B. licheniformis* cells on sodium phytate (Sigma-Aldrich Co, USA) as an alternative phosphate source was studied in a phosphate limited BMM by the addition of 0.5 mM sodium phytate 2 h after onset of the stationary growth phase. Growth experiments were done in 50 mL BMM in 250 mL Erlenmeyer flasks at 37 °C under vigorous at 200 rpm. The growth of the *B. licheniformis* strains was determined by measuring the optical density (OD) of the cultures every two hours at a wavelength of 500 nm.

In order to elucidate whether phytate is an inducer of the P*phyL* promoter, *B. licheniformis* strains carrying the translational fusion of P*phyL*′-′*amyE* and P*phyL*′-′*xynA* were cultivated in a phosphate limited BMM with sodium phytate, which was added to the growth medium at an OD (at 500 nm) of 1.0 with the final concentrations of either 0.5 mM or 5 mM. The cell samples were taken during the logarithmic growth phase at an OD of 1.0 and 1, 2, 3 and 4 h after onset of the stationary growth phase.

### RNA isolation and northern blot analysis

Cell disruption was performed by using the RiboLyser Cell Disrupter (Thermo Electron Corporation, Germany) and total RNA was isolated and purified by using the KingFisher mL pipetting robot (Thermo Electron Corporation, Germany) by means of the MagNA Pure LC RNA isolation Kit I (Roche Diagnostics, Germany) as described in detail by Jürgen et al. [24]. The quality of the isolated total RNA was analyzed by means of the Bioanalyzer 2100 from Agilent (Germany).

The effect of phytate on the expression of the P*phyL* controlled expression of the heterologous *amyE* gene was determined by Northern blot analyses as described by Wetzstein et al. [25]. The specific hybridization reaction for the *amyE* mRNA was performed with appropriate digoxigenin-labeled RNA probes. The probes were synthesized with the T7 RNA polymerase from the T7 promoter-containing internal PCR products of the *amyE* gene using the primer pairs 8/9 (Additional file 1: Table S1).

## Results

### Analysis of the *phyL* promoter sequence

**Fig. 1:**
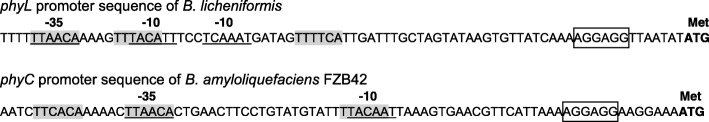
Comparison of the promoter regions of the *phyL* gene from *B. licheniformis* and the *phyC* gene from *B. amyloliquefaciens* FZB42. Consensus sequences (− 35 and − 10) are underlined. The boxes indicate the putative ribosome binding sites. Grey shading indicates the putative PhoP binding boxes. The structure of the *phyC* promoter of *B. amyloliquefaciens* FZB42 was investigated in detail by Makarewicz et al. [26]

A recent transcriptome analysis indicated a phosphate controlled expression of the *phyL* gene of *B. licheniformis* (Additional file 1: Figure S1) [11]. In addition, the study of Hoi et al. [11] also revealed that phytase is one of the most prominent proteins in the secretome of *B. licheniformis* cells under phosphate limited growth conditions. In order to elucidate the structure of the *phyL* promoter of *B. licheniformis* the predicted promoter region with the well-known promoter sequence of the phytase encoding gene *phyC* from *Bacillus amyloliquefaciens* FZB42, a close relative of *B. licheniformis* was aligned. It is shown that the *phyL* promoter region contains a σ^A^-like promoter sequence, which well resembles the reported − 10 and − 35 sequences of the *phyC* promoter (Fig. 1). In addition, the *phyL* promoter region contained another − 10-like consensus sequence, which is located between the putative − 10 and − 35 promoter sequence (Fig. 1). A pair of tandemly repeated PhoP TT(T/A/C)ACA binding boxes was found within and downstream of the − 35 consensus promoter sequence. Another putative PhoP binding box was located downstream of the − 10 consensus region of the *phyL* promoter. This suggests that the expression of the *phyL* gene in *B. licheniformis* is probably regulated by the PhoPR two component system as reported for the *phyC* gene in *B. amyloliquefaciens* FZB45 (Fig. 1).

### P*phyL* controlled expression patterns of the *amyE* and *xynA* genes

**Fig. 2:**
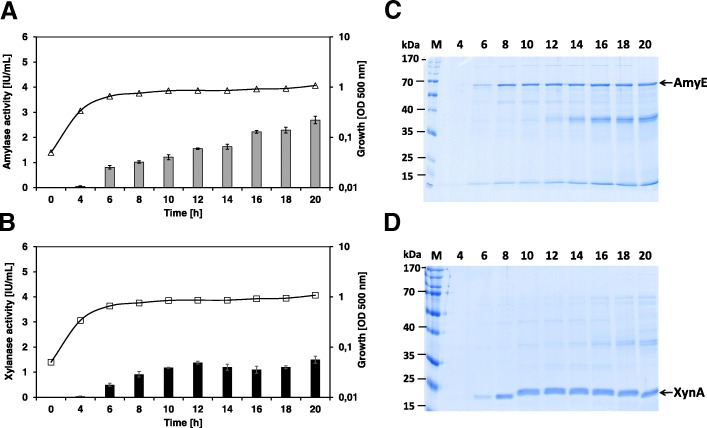
The *phyL* promoter-dependend expression of the recombinant amylase and xylanase encoding genes of the *B. licheniformis* strains TH3 (**a**) and TH4 (**b**) under phosphate limited growth conditions (*n* = 3 independent cultivations). *Lines* indicate cell growth, while *bars* indicate enzyme activity. Growth: *triangles* TH3, *squares* TH4. Enzyme activity: *grey bars* amylase, *black bars* xylanase. **c** and **d** are SDS-PAGE separations of extracellular proteins in *B. licheniformis* strains TH3 and TH4, respectively. Each sample contained 20 μL of culture medium after removal of the cells by centrifugation. Protein bands are indicated by arrows

The phosphate regulated promoter, P*phyL*, was cloned upstream of the reporter genes *amyE* and *xynA*, resulting in the plasmids pKUC3 (strain TH3) and pKUC4 (strain TH4) respectively. The expression pattern of the *B. licheniformis* strains containing one of those plasmids revealed that both promoter fusions started to express the *amyE* and *xynA* reporter genes when cells entered the transient phase (6 h after inoculation) in a phosphate limited BMM (Fig. 2). In order to verify that the synthetic constructs of the *phyL* promoter and the reporter genes are indeed mainly phosphate-dependent regulated during the onset of the stationary phase, the strain *B. licheniformis* TH3, carrying the P*phyL-amyE* fusion, was exemplarily cultivated with different phosphate concentrations (0.15, 0.3 and 0.6 mM) (Additional file 1: Figure S3). The data demonstrate that α-amylase activity was significantly increased under phosphate-limited conditions (0.15 mM). In contrast, only low α-amylase activities were detectable in cultivations with high phosphate concentrations. Furthermore, no amylase or xylanase activity was detected during the exponential phase without phosphate limitation. The amylase activity in the P*phyL*′-′*amyE* fusion strain TH3 increased gradually throughout the stationary phase and reached a final maximal activity of about 2.5 IU/mL (Fig. 2a). The P*phyL*′-′*xynA* fusion strain TH4 showed a similar final xylanase activity throughout the stationary phase of about 1.5 IU/mL (Fig. 2b).

The SDS-PAGE analysis of the extracellular protein fraction of the strain TH3 revealed that the heterologous AmyE protein started to secrete and accumulate in the extracellular medium at the transient phase (6 h after cultivation). The AmyE level increased gradually throughout the stationary phase (Fig. 2c). The production pattern of the heterologous XynA protein in strain TH4 was similar to the production pattern observed for the AmyE protein (Fig. 2d).

### Analysis of phytate as alternative substrate and inducer

**Fig. 3:**
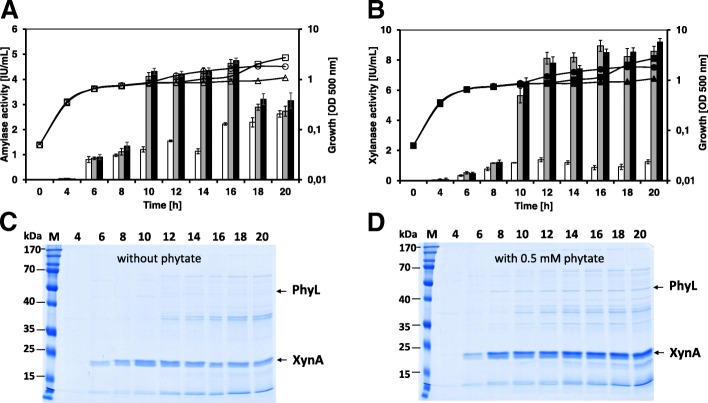
The expression of the recombinant amylase and xylanase driven by the *phyL* promoter (*n* = 3 independent cultivations) of the *B. licheniformis* strains TH3 (**a**) and TH4 (**b**) without and with the addition of sodium phytate. Sodium phytate was added at an OD_500 nm_ of 1.0 (after 8 h cultivation). *Lines* indicate cell growth, while *bars* indicate enzyme activity. Growth: *Open symbols* TH3, *filled symbols* TH4; *triangles* without the addition of sodium phytate, *circles* with 0.5 mM sodium phytate, *squares* with 5 mM sodium phytate. Enzyme activity: *white bars* without the addition of sodium phytate, *grey bars* with 0.5 mM sodium phytate, *black bars* with 5 mM sodium phytate. SDS-PAGE separation of extracellular proteins in 20 μL of culture supernatant of strain TH4 without addition of sodium phytate (**c**) and with 0.5 mM sodium phytate (**d**). Sodium phytate was added at an OD _500 nm_ of 1.0 (after 8 h cultivation). Protein bands are indicated by arrows

*B. licheniformis* was then tested for its ability to use phytate as alternative phosphate source when cells were subjected to phosphate limitation. The growth of *B. licheniformis* cells was monitored in phosphate-limited BMM in the absence or presence of sodium phytate. In growth experiments without the addition of sodium phytate, *B. licheniformis* cells entered the transient phase after 6 h of cultivation at an OD _500 nm_ of about 0.8. The growth rate of the control and the phytate containing culture remained constant until an OD of around 1.0 was reached. However, in the culture with 0.5 mM sodium phytate the OD values increased gradually up to an OD of about 2. When higher amounts of sodium phytate (5 mM) were added to the growth medium, *B. licheniformis* cells grew more slowly to an OD value of 1.0 within 8 h, however, the biomass significantly increased up to an OD value of 2.7 at the end of the cultivation (Fig. 3).

**Fig. 4:**
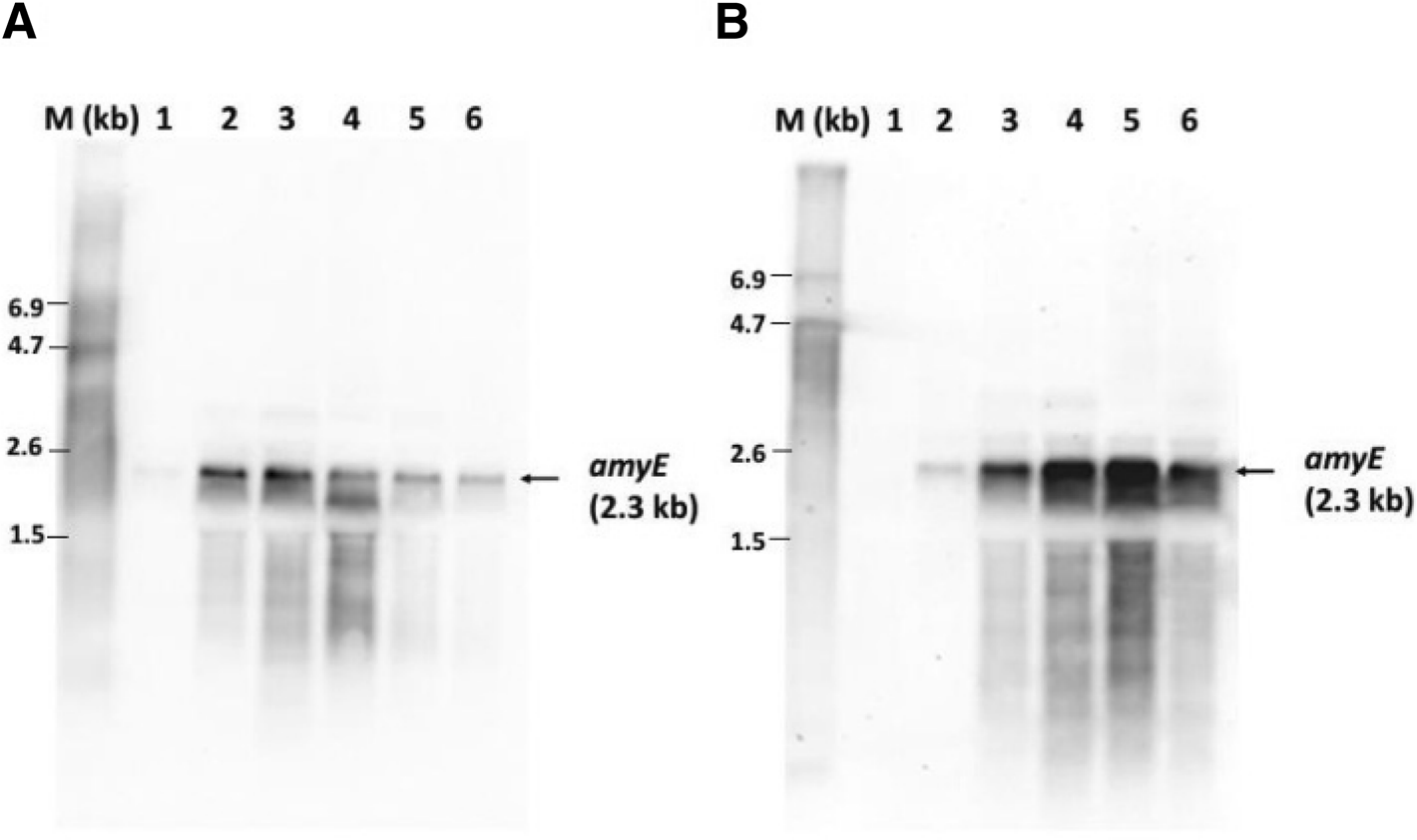
Northern-blot analysis of the *amyE* gene expression of the *B. licheniformis* strain TH3 without (**a**) and with (**b**) 0.5 mM sodium phytate (added at an OD_500 nm_ of 1.0). RNA samples were isolated from exponentially growing cells at an OD of 0.4 (1), at an OD of 1.0 (2) and 1, 2, 3 and 4 h after an OD of 1.0 (3, 4, 5 and 6). 10 μg of total RNA was used for each sample

In order to investigate whether phytate might serve as an inducer of the *phyL* promoter, the *B. licheniformis* strains TH3 and TH4 bearing the P*phyL*′-′*amyE* and P*phyL*′-′*xynA* fusions, were cultivated in phosphate limited BMM supplemented with different concentrations of sodium phytate. Under growth conditions without sodium phytate, the P*phyL*′-′*amyE* fusion strain TH3 showed a maximal amylase activity of about 2.6 IU/mL, while the P*phyL*′-′*xynA* fusion strain TH4 reached a maximal xylanase activity of about 1.5 IU/mL (Fig. 3a and b). When 0.5 mM sodium phytate was added to the growth medium, the *phyL*′-′*amyE* fusion strain TH3 reached a higher maximal amylase activity of 4.5 IU/mL (Fig. 3a). This observation was supported by Northern blot analyses of the strain TH3, which revealed a significant higher *amyE* transcript level under growth conditions with 0.5 mM sodium phytate compared to the control without phytate addition (Fig. 4). The addition of 0.5 mM phytate to the growth medium of the *phyL*′-′*xynA* fusion strain TH4 resulted in higher maximal xylanase activity of 9.2 IU/mL (Fig. 3b). Such an increase in the expression pattern could also be observed for the XynA protein level in strain TH4 under growth conditions with 0.5 mM phytate (Fig. 3d). The application of a higher phytate concentration (5 mM) did not result in higher amylase or xylanase activities (Fig. 3a and b). It is worth mentioning that the native phytase protein PhyL was overproduced and secreted when phytate was added to the phosphate limited BMM (Fig. 3d).

## Discussion

A global transcriptome and proteome analysis revealed that more than 100 genes are significantly upregulated in *B. licheniformis* in response to phosphate limitation [11]. The *phyL* transcript belonged to the most strongly induced and abundant mRNAs during the transition phase from the exponential to stationary phase. A sequence analysis indicates that the *phyL* gene is similar to other phosphate-controlled genes regulated by the PhoPR two component system. Furthermore, the − 10 and − 35 regions of the *phyL* promoter reveal typical SigmaA-dependent sequences (Fig. 1). Putative PhoP binding boxes were found within and downstream of the *phyL* promoter region. Such a specific PhoP control was recently shown for the highly similar *phyC* promoter of the closely related species *B. amyloliquefaciens* [26]. Data of this study reveal that the *phyL* promoter was only induced when cells are exposed to phosphate limitation. As suggested by Makarewicz et al. [26], both Eσ^A^ RNAP holoenzyme and PhoP~P are necessary and sufficient to establish the transcriptional activation of the *phyC* promoter in *B. amyloliquefaciens* under such growth conditions. Thus, the expression of the *phyL* promoter in *B. licheniformis* might be similarly regulated as described for the *phyC* promoter in *B. amyloliquefaciens*.

Data of this study indicate an efficient expression of two heterologous model proteins by using the tightly regulated and strongly inducible P*phyL* promoter in *B. licheniformis* under phosphate-limitation conditions. Neither protein bands in a 1D-SDS-PAGE nor activities of the two model proteins, the amylase or the xylanase, could be detected during the exponential growth phase. Furthermore, the growth behavior of the recombinant strains also indicates that perturbing effects of the proposed expression system on the growth of *B. licheniformis* cells during the exponential growth phase, can be excluded as long as sufficient inorganic phosphate is available. In addition, it is shown that sodium phytate is a suitable alternative phosphate source for the growth of *B. licheniformis* when cells were subjected to phosphate limited growth conditions. Experiments in this study suggest that moderate concentrations of sodium phytate (≤ 5 mM) would be more favorable to induce the activity of the *phyL* promoter in *B. licheniformis*. The addition of higher concentrations of phytate (e.g. 1% *w*/*v* or 15 mM) to the growth medium could hamper the activity of the promoter of the phytase gene in *B. licheniformis* (data not shown). This could be due to a critical increase of inorganic phosphate levels by the phytate hydrolysis of the induced phytase enzyme, which would down-regulate the activity of the phosphate responsive PhoPR two-component system and thus result in a lower P*phyL* activity.

For *B. subtilis* a similar phosphate controlled expression system based on the *pst* promoter was suggested [10]. However, a crucial starvation of an essential substrate, such as phosphate, during the protein over-production phase could diminish the protein synthesis capacity of the host. Therefore, to reach optimal yields, either the promoter of an appropriate expression system has to be switched on before the complete exhaustion of the critical substrate or the limited nutrient needs to be replaced by another suitable substrate, which does not lead to a down-regulation of the promoter system. Thus, the perfect alternative substrate should be metabolized and in parallel be an inducer of the system [9]. Data of this study indicated that the expression system using the *phyL* promoter is not only strong and tightly regulated by the level of inorganic phosphate but also easily inducible by the alternative phosphate source phytate. The suggested promoter system is comparable to the *pst* promoter system of *B. subtilis* [10] but exhibits an additional feature due to its expression stimulation by the alternative phosphate source phytate.

## Conclusions

The results of this study demonstrate that the *phyL* promoter is a suitable candidate for an auto-inducible expression system for *B. licheniformis*. It is shown that phytate is not only an appropriate alternative phosphate source for this bacterium, but also an inducer of this expression system. The P*phyL* expression system might be used to overexpress target genes in *B. licheniformis* under growth conditions, which can be easily performed in industrial batch-fermentation processes. However, further studies are required to investigate in detail the suitability of this auto-inducible system for large-scale heterologous protein production in *B. licheniformis* fed-batch fermentation processes.

## Additional files

Additional file 1: **Figure S1.** Heat map of expression levels of selected phosphate starvation inducible genes in *B. licheniformis* [11]. Low and high values are given in blue with different gradients. **Figure S2.** The 323 bp large DNA-sequence of the phytase promoter (P*phyL*), which was used to express *amyA* and *xynA*. **Figure S3.** The *phyL* promoter driven expression of the recombinant amylase (*n* = 3 independent cultivations) of *B. licheniformis* TH3 in BMM supplemented with different concentrations of phosphate. *Lines* indicate cell growth, while *bars* indicate enzyme activity. Growth: *triangles* with 0.15 mM phosphate, *circles* with 0.3 mM phosphate, *squares* with 0.6 mM phosphate. Enzyme activity: *grey bars* with 0.6 mM phosphate, *black bars* with 0.3 mM phosphate, *white bars* with 0.15 mM phosphate. **Table S1.** Sequences of primers used in this study. (DOCX 50 kb)

## Abbreviations

APS: Ammonium persulfate
BMM: Belitzky minimal medium
IU: International unit
OD: Optical density
PAGE: Polyacrylamide gel electrophoresis
Pi: Inorganic phosphate
RNAP: RNA polymerase
SDS: Sodium dodecyl sulfate
TEMED: Tetramethylethylenediamine
