# Supplementary material for: An auto-inducible phosphate-controlled expression system of Bacillus licheniformis
Source: BMC Biotechnol. 2019 Jan 9;19:3. doi: 10.1186/s12896-018-0490-6 (PMC6327384; doi:10.1186/s12896-018-0490-6)
Supplement: Supplementary file 1 — Figure S1. Heat map of expression levels of selected phosphate starvation inducible genes in B. licheniformis [11]. Low and high values are given in blue with different gradients. Figure S2. The 323 bp large DNA-sequence of the phytase promoter (PphyL), which was used to express amyA and xynA. Figure S3. The phyL promoter driven expression of the recombinant amylase (n = 3 independent cultivations) of B. licheniformis TH3 in BMM supplemented with different concentrations of phosphate. Lines indicate cell growth, while bars indicate enzyme activity. Growth: triangles with 0.15 mM phosphate, circles with 0.3 mM phosphate, squares with 0.6 mM phosphate. Enzyme activity: grey bars with 0.6 mM phosphate, black bars with 0.3 mM phosphate, white bars with 0.15 mM phosphate. Table S1. Sequences of primers used in this study. (DOCX 50 kb) [file 12896_2018_490_MOESM1_ESM.docx]

**Trung et al. - Supplementary online material**

**Fig. S1.**

**Fig. S2**

atccatcctgctcgggatcattttgatgctgctgtcaccgaaaattcagaaatttatgaagggtgttaattaaaaagcaataggctgatactccttttcttctgttttttcgcaggatatatacatttcgatttaatgagagatagggaatacctgttaaagcaaaacccccggaaagaattccgggggttttctctgcggcttcgtatgctaaatctcacagcccctttaaaccaccgcttttttaacaaaagtttacatttcctcaaatgatagttttcattgatttgctagtataagtgttatcaaaaggaggttaatat

**Fig. S3**

**
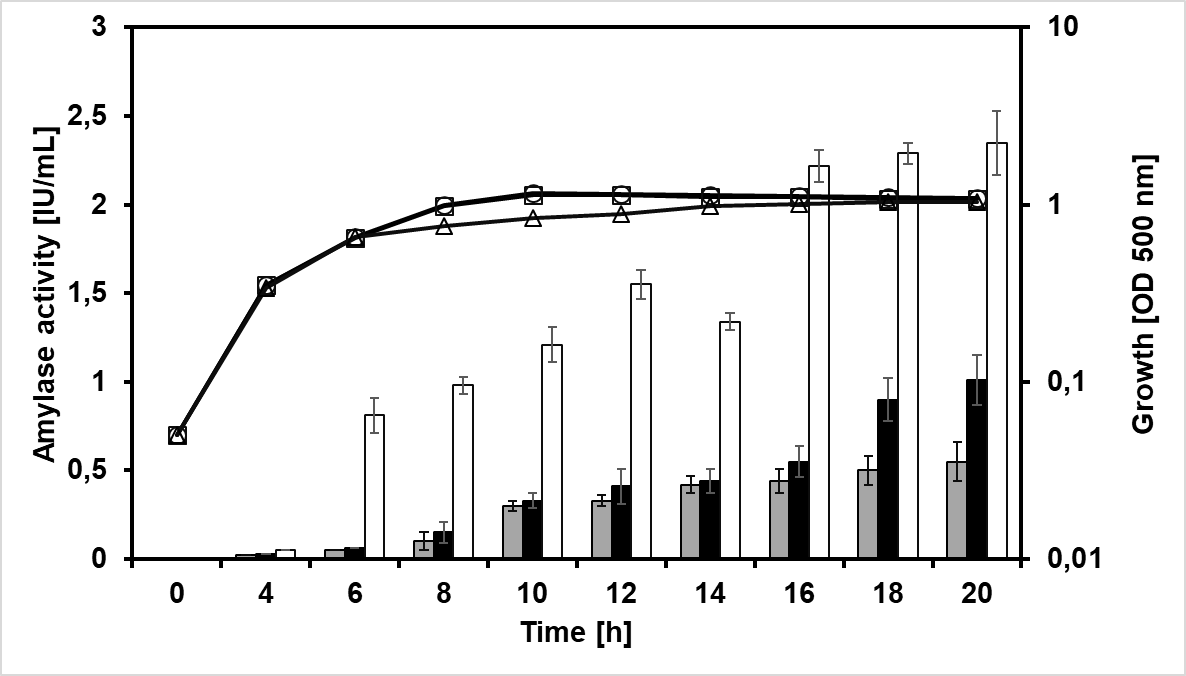
**

**Table S1.** **Sequences of primers used in this study.**

| **Primer number** | **Sequence 5´-3´** |
| --- | --- |
| 1 | GAGTATCTAGAATCCATCCTGCTCGGGATC |
| 2  3  4  5  6  7 | GAATCGTTTTGCAAACATATATTAACCTCC  GGAGGTTAATATATGTTTGCAAAACGATTC  GATGTAAGGCGGTGGATACATGTTTG  GTTTTTTTTAAATTTAAACATATATTAACCTCCTTTTG  CAAAAGGAGGTTAATATATGTTTAAATTTAAAAAAAAC  GTCGAGGTACCATAGAAAAAGAGCATTTTTTG |
| 8  9 | GATGCAGGATATACAGCCATTCAG  CTAATACGACTCACTATAGGGAGACGTTTCAGATAGGACTGTAC |
